# Zinc restrains multicellular remodeling in fibrotic lung disease

**DOI:** 10.1172/jci.insight.210198

**Published:** 2026-09-15

**Authors:** Jianfei Ji, Wenjing You, Xiaoli Sun, Peng Zhao

**Affiliations:** 1 Department of Biochemistry and Structural Biology,; 2 Department of Pharmacology, and; 3 Transplant Center, The University of Texas Health Science Center San Antonio, San Antonio, Texas, USA.

**Keywords:** Immunology, Pulmonology, Fibrosis, Macrophages, Transcriptomics

## Abstract

Zinc restrains multicellular remodeling and attenuates fibrogenesis in fibrotic lung disease

**To the Editor:** Idiopathic pulmonary fibrosis (IPF) is a progressive lung disease featured by extensive tissue remodeling with altered functions of epithelial, endothelial, mesenchymal, and immune cells. This multicellular dysfunction is a central driver of disease progression, yet the factors that shape these coordinated changes remain poorly understood. Zinc is an essential micronutrient reported to regulate redox balance, epithelial integrity, and immune homeostasis (1). A previous study reported that zinc transporter regulates alveolar progenitor cell renewal during lung injury (2). However, whether and how zinc modulates functions of different cell types and whether dietary zinc affects IPF-associated pathogenic pathways remain unclear.

To evaluate the effects of dietary zinc on injury-associated pulmonary fibrosis, we used bleomycin to induce lung injury, and we fed mice with a normal-, low-, or high-zinc diet. H&E and Sirius red staining showed that a high-zinc diet attenuated pulmonary fibrosis and preserved normal lung morphology. In contrast, a low-zinc diet exacerbated pulmonary injury and fibrosis (Figure 1A). Moreover, a high-zinc diet significantly increased O_2_ saturation in bleomycin-treated mice, indicating improved lung function, while a low-zinc diet exhibited opposite effects (Figure 1B). Analyses of O_2_ consumption (VO_2_) and CO_2_ excretion (VCO_2_) further showed that high-zinc diet–fed mice maintained higher VO_2_ and VCO_2_, suggesting better preserved gas exchange functions (Figure 1C). Together, these data indicate that zinc plays an important role in preventing pulmonary injury and fibrosis.

To define the cellular basis of zinc-associated protection against lung fibrosis, we conducted single-cell RNA-seq (scRNA-seq) to profile pulmonary cells in control mice (no lung injury) and bleomycin-treated mice fed a normal-, low-, or high-zinc diet. UMAP visualization identified major lung epithelial, endothelial, mesenchymal, and immune cell populations, including alveolar type 1 (AT1) cells, AT2 cells, transitional AT1/2 cells, capillary endothelial cells, fibroblasts, myofibroblasts, pericytes, alveolar macrophages (AMs), interstitial macrophages (IMs), monocytes, neutrophils, lymphocytes, and dendritic cells (Figure 1D and Supplemental Figure 1A; supplemental material available online with this article; https://doi.org/10.1172/jci.insight.210198DS1). Compared with control mice, bleomycin-treated mice showed altered pulmonary cell composition, with an increase of IMs and transitional AT1/2 cells (Figure 1D and Supplemental Figure 1, B–D). This observation is consistent with previous reports on pulmonary macrophage remodeling and stalled AT2-to-AT1 transition in IPF (3, 4). Importantly, the pathological increases of IMs and AT1/2 cells were accentuated in mice fed a low-zinc diet and largely attenuated by a high-zinc diet (Figure 1D).

Furthermore, transcriptomic analysis revealed that bleomycin induced a multicellular transcriptional remodeling, while a high-zinc diet significantly reversed these changes (Figure 1E). In IMs, a high-zinc diet prevented bleomycin-induced upregulation of *Spp1*, *Cd9*, *Lgals3*, *Gpnmb*, *Acp5*, *Ctss*, *Ctsd*, *Ctsb*, *Ctsl*, *Fn1*, *Thbs1*, *Mmp12*, and *Mmp14* — genes contributing to pulmonary fibrosis. In myofibroblasts, the cell type directly mediating fibrosis, a high-zinc diet downregulated genes involved in extracellular matrix (ECM) remodeling, including *Timp1*, *Spp1, Col1a1*, *Col3a1*, *Fn1*, *Tnc*, *Sparc*, *Thbs1*, *Lox*, *Loxl2*, *Fkbp10*, *Ltbp2*, *Ccn2*, *Acta2*, *Tagln*, and *Cald1*. In contrast, a low-zinc diet maintained or further enhanced expression of these genes (Figure 1F and Supplemental Figure 2). Intriguingly, a high-zinc diet markedly reversed bleomycin-induced transcriptional changes in AT1, AT2, and AT1/2 cells, including genes associated with ECM remodeling (*Col1a1*, *Col1a2*, *Fn1*, *Spp1*, and *Mmp12*), inflammation (*Serpina3n*), and DNA damage-induced cell cycle arrest (*Cdkn1a*) (Figure 1G and Supplemental Figure 1E). Gene set enrichment analysis (GSEA) further revealed a conserved transcriptomic signature across human IPF (GSE136831) and bleomycin-induced lung fibrosis. In humans, IPF was associated with broad enrichment of angiogenesis, coagulation, epithelial-mesenchymal transition, collagen assembly, collagen biosynthesis, and ECM organization pathways across multiple cell types. A similar transcriptomic remodeling was induced by bleomycin in mice. Notably, a high-zinc diet suppressed the activation of these IPF-associated pathological pathways (Figure 1H). SPP1^+^ IMs were previously reported to promote pulmonary fibrosis (5, 6). Our analysis of a human dataset confirmed that SPP1^+^ IMs were enriched in IPF lungs (Supplemental Figure 3). Bleomycin induced a similar increase of SPP1^+^ IMs. This effect was diminished by a high-zinc diet (Figure 1I). Immunofluorescence staining confirmed that bleomycin significantly increased SPP1^+^ macrophages (SPP1^+^CD68^+^), while a high-zinc diet markedly attenuated this increase (Figure 1, J and K). Analyses of chemokine expression across different cell types showed that bleomycin significantly upregulated *Ccl8*, a chemokine associated with IPF prognosis (7), in myofibroblast. This induction was largely attenuated by a high-zinc diet (Figure 1L), suggesting that myofibroblast-derived chemotactic signal contributes to macrophage recruitment. Together, these findings indicate that a high-zinc diet not only attenuates fibrosis but also broadly restrains multicellular remodeling associated with human IPF.

Our studies indicate that zinc prevents pathological tissue remodeling in injury-associated lung fibrosis. Rather than acting on a single effector, a high-zinc diet broadly restrained disease-associated multicellular remodeling, including SPP1^+^ IM expansion, myofibroblast activation, transitional AT1/2 epithelial cell accumulation, and profibrotic gene upregulation. Notably, the zinc-responsive transcriptional changes converged with pathways activated in human IPF, highlighting the translational relevance of our findings. In contrast, a low-zinc diet exacerbated these pathological changes, underscoring the sensitivity of lung fibrotic remodeling to zinc availability. These results extend prior work linking zinc transporter ZIP8 to alveolar progenitor renewal and place zinc biology within a broader multicellular network of IPF pathogenesis. The precise mechanisms by which zinc restrains multicellular remodeling and whether zinc supplementation benefits established fibrosis remain important questions for future investigation. Together, our findings suggest that dietary zinc broadly reprograms pulmonary cell functions and protects against lung fibrosis.

## Conflict of interest

The authors declared that no conflict of interest exists.

## Funding support

This work was supported by the following:

- Cancer Prevention and Research Institute of Texas (CPRIT) RR210005 (XS) and RR200089 (PZ).
- Voelcker Young Investigator Award AWD0000144 (PZ).

## Supplementary Material

Supplemental data

Supporting data values

## Figures and Tables

**Figure 1:**
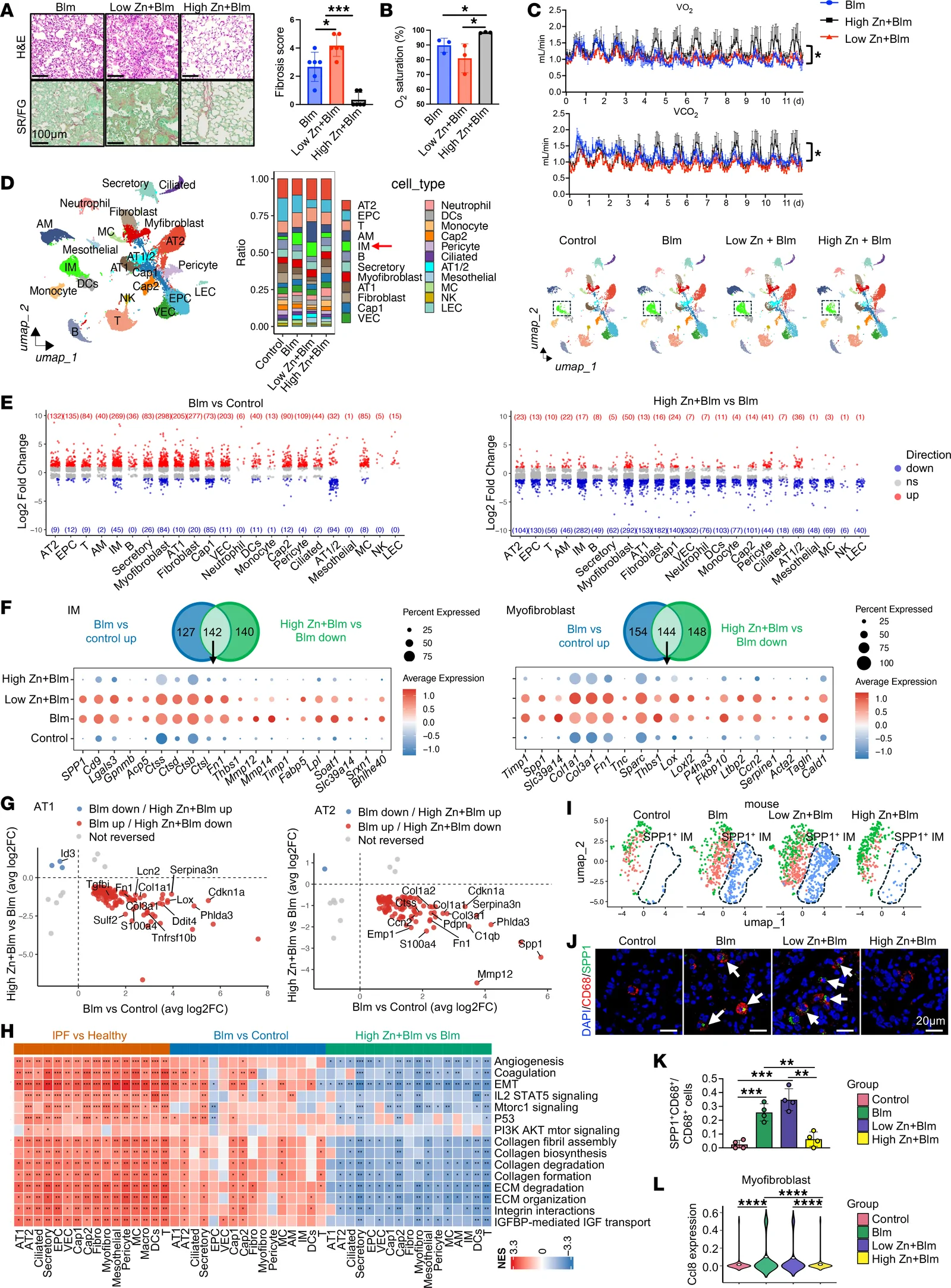
Dietary zinc regulates bleomycin-induced lung fibrosis and multicellular remodeling. Bleomycin-treated mice were fed diets containing normal, low, or high zinc. (**A**) H&E and Sirius red staining of lung sections showing tissue morphology and fibrosis. Scale bar: 100 μm. Quantification of fibrosis (right). *n* = 6. Unpaired 2-tailed *t* test. (**B**) Oxygen saturation rate of indicated mice. *n* = 3. Unpaired 2-tailed *t* test. (**C**) Oxygen consumption (VO_2_), and carbon dioxide excretion (VCO_2_) of indicated mice. *n* = 4. ANCOVA. (**D**) UMAP annotation of lung scRNA-seq data (left). Quantification of cell populations (middle). UMAP showing cell populations (square: IMs) (right). (**E**) Scatter plots showing differentially expressed genes with |log_2_ FC| > 1 and *P*_adj_ < 0.05. (**F**) Overlap between genes upregulated by bleomycin (Blm) and genes downregulated by a high-zinc diet in IMs and myofibroblasts. (**G**) Expression of genes significantly regulated by Blm in AT1 and AT2 cells. (**H**) Pathway heatmap comparing scRNA-seq data (GSE136831) from human IPF lungs (Healthy, 28; IPF, 32), mouse Blm versus Control, and High Zinc + Blm (Zn+Blm) versus Blm lungs. (**I**) UMAP (annotated in Supplemental Figure 3D) showing SPP1^+^ IM in scRNA-seq data. (**J**) IF staining of CD68 and SPP1. Arrows indicate SPP1^+^CD68^+^ cells. Scale bar: 20 μm. *n* = 4. (**K**) Ratio of CD68^+^SPP1^+^/CD68^+^cells. Unpaired 2-tailed *t* test. (**L**) *Ccl8* expression in myofibroblast. Wilcoxon rank-sum test. *P < 0.05, **P < 0.01, ***P < 0.001, ****P < 0.0001.

## Version 1 09/15/2026

In-Press Preview

## Version 2 10/22/2026

Electronic publication
