# Supplementary material for: Zinc restrains multicellular remodeling in fibrotic lung disease
Source: JCI Insight. 2026 Sep 15;11(20):e210198. doi: 10.1172/jci.insight.210198 (PMC13726553; doi:10.1172/jci.insight.210198)
Supplement: Supplementary file 1 [file jciinsight-11-210198-s002.pdf]

Zinc Restrains Multicellular Remodeling in Fibrotic Lung Disease

Jianfei Ji[1,2], Wenjing You[1,2], Xiaoli Sun[2,3], Peng Zhao[1,*]

Supplemental Materials

Supplemental Figures

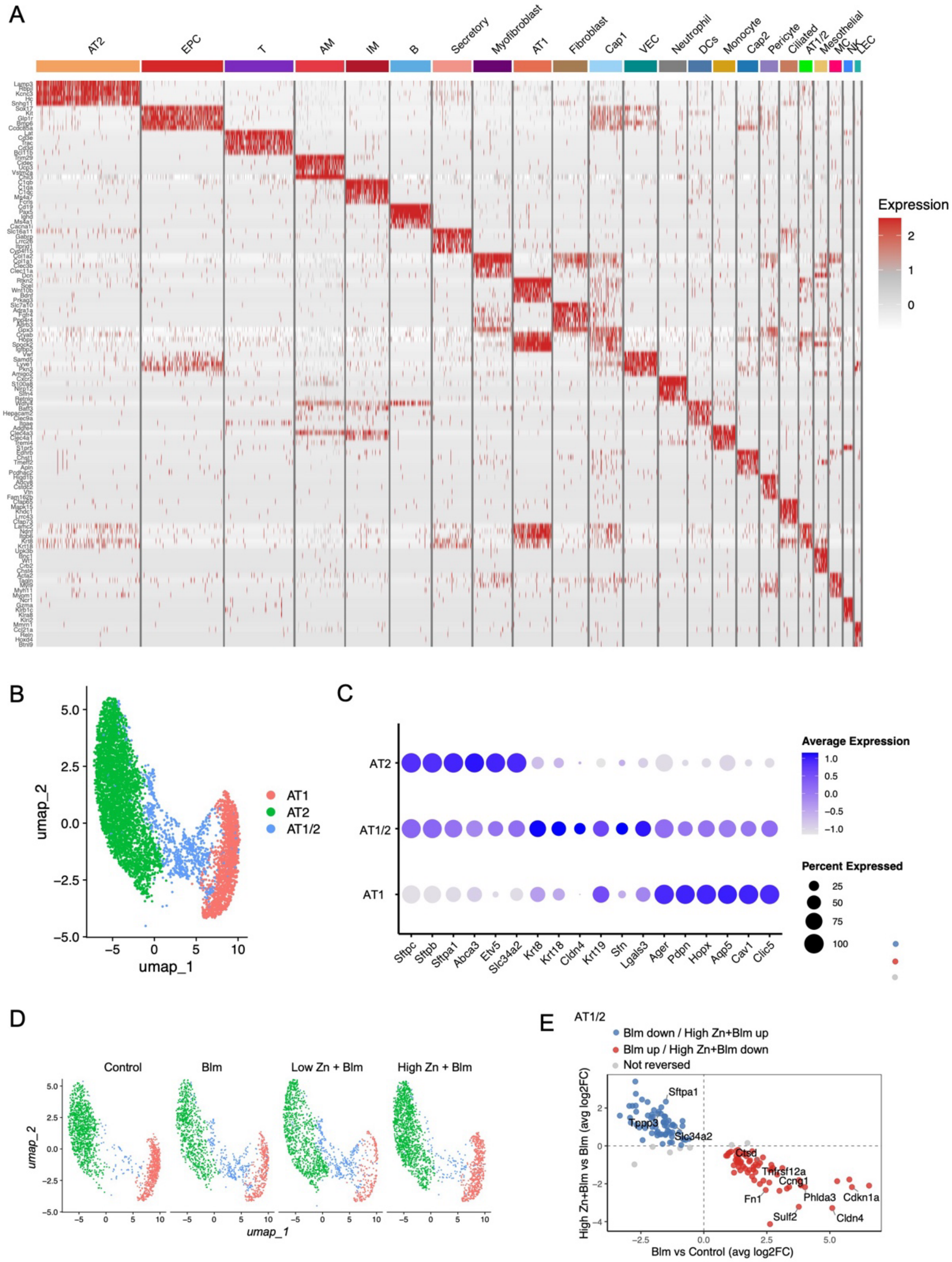

Figure S1. Annotation of major pulmonary cell populations in mouse lung scRNA-seq data. **(A)** Heatmap showing the expression of the top 5 marker genes for each annotated pulmonary cell population in scRNA-seq. Cell types were annotated based on canonical lineage markers. **(B)** UMAP visualization of AT1, AT2, and transitional AT1/2 cells. **(C)** Dot plot showing canonical markers of AT1, AT2, and transitional AT1/2 cells. Dot size represents the percentage of cells expressing each gene, and color indicates average expression. **(D)** UMAP visualization of alveolar epithelial populations across the indicated groups. **(E)** Expression of genes significantly regulated by Blm in AT1/2 cells.

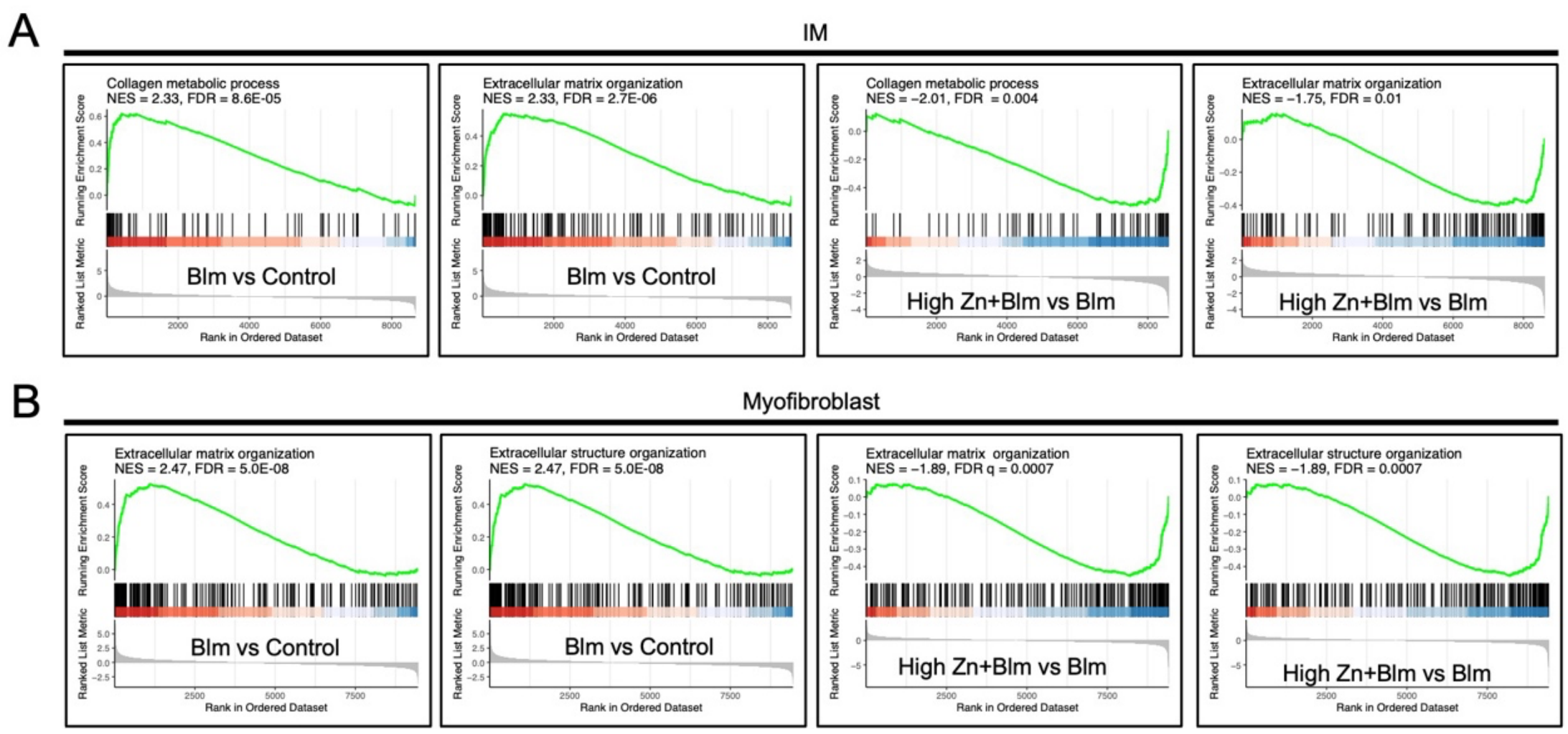

Figure S2. High-zinc diet attenuates bleomycin-induced transcriptional and profibrotic pathway across lung cell populations. **(A)** GSEA plots of genes upregulated by bleomycin and downregulated by high-zinc diet showing enrichment of collagen metabolic process and extracellular matrix organization in interstitial macrophages. **(B)** GSEA plots of genes upregulated by bleomycin and downregulated by high-zinc diet showing enrichment of extracellular matrix organization and extracellular structure organization pathways in myofibroblasts.

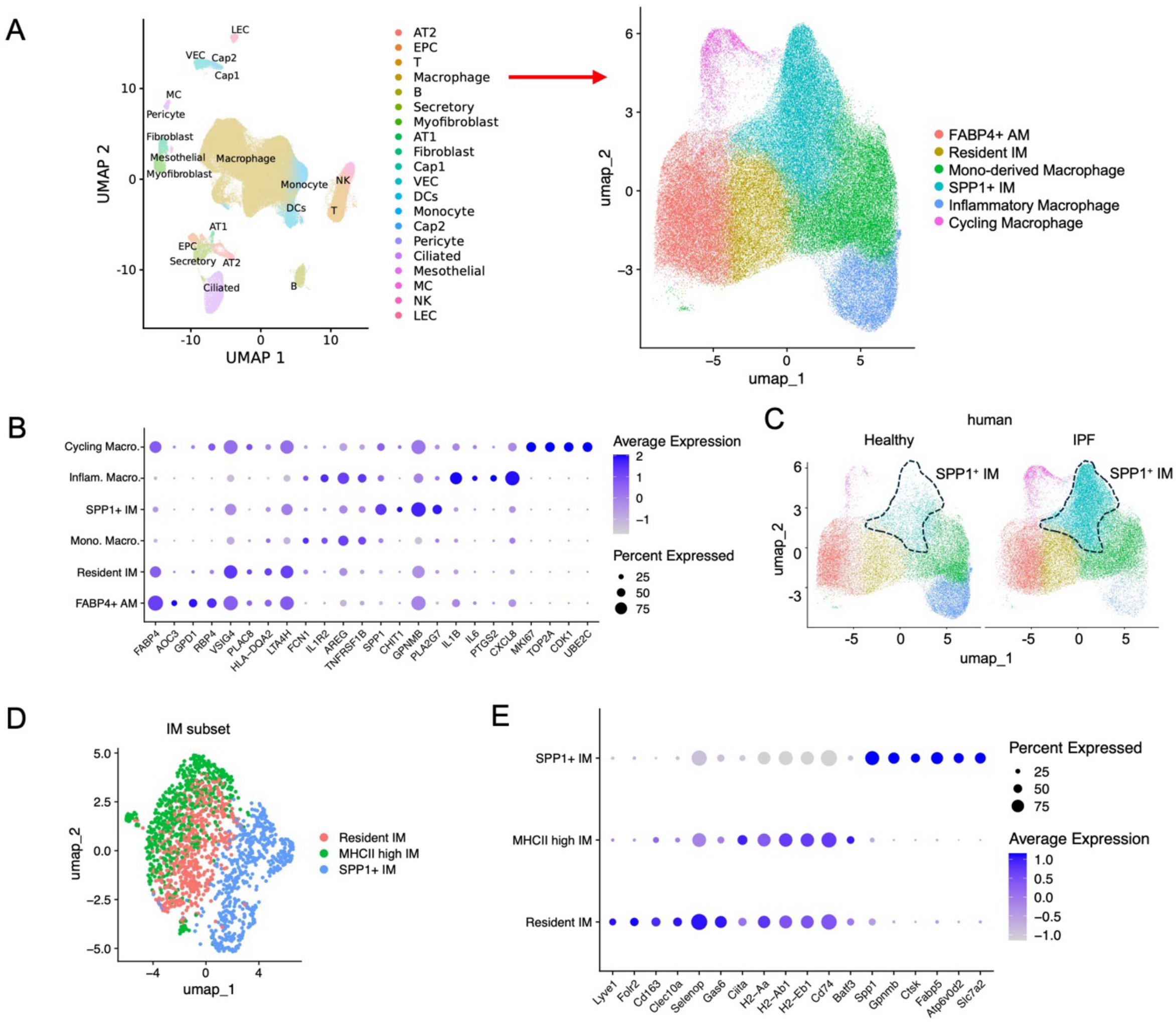

Figure S3. Identification of SPP1⁺ interstitial macrophages in human IPF and mouse with bleomycin-induced lung injury. (A) UMAP visualization of human lung scRNA-seq data (GSE136831) from human IPF lungs (Healthy = 28, IPF = 32). Major pulmonary cell populations were annotated in the integrated dataset, and macrophages were further separated and re-clustered to define macrophage subpopulations. **(B)** Dot plot showing marker gene expression across human macrophage subpopulations, including FABP4⁺ alveolar macrophages, resident interstitial macrophages, monocyte-derived macrophages, SPP1⁺ interstitial macrophages, inflammatory macrophages, and cycling macrophages. Dot size indicates the percentage of cells expressing each gene, and color indicates average expression. **(C)** UMAP plots showing the distribution of SPP1⁺

interstitial macrophages in healthy and IPF lungs from human dataset GSE136831. The dashed outline highlights the SPP1$^+$ IM population, which is expanded in IPF lungs comparing to healthy controls. **(D)** UMAP visualization of mouse interstitial macrophage subclusters from lungs of bleomycin-administered mice, including resident IMs, MHCII high IMs, and SPP1$^+$ IMs. **(E)** Dot plot showing marker gene expression across subtypes of mouse IMs.

Methods

Mouse models

C57BL/6J mice were purchased from The Jackson Laboratory. Mice were housed within pathogen-free animal facility with a 12-hour light, 12-hour dark cycle, and given free access to food and water. All animal procedures were approved by the Institutional Animal Care and Use Committee (IACUC) of The University of Texas Health Science Center at San Antonio. All procedures involving mice were conducted in accordance with the Guide for Care and Use of Laboratory Animals of the NIH (National Academies Press, 2011). 16 weeks old mice were randomly assigned to zinc-defined diets before bleomycin-induced lung injury. Mice were fed a low-, normal-, or high-zinc diet containing 20 ppm, 80 ppm, or 120 ppm zinc, respectively, for 2 weeks before bleomycin administration and maintained on the same diets throughout the experiment (1). Lung fibrosis was induced by intranasal administration of bleomycin at 1.5 U per mouse under anesthesia. Control mice received vehicle. Mice were monitored after bleomycin challenge, and lung tissues were collected 14 days after bleomycin administration for histological analysis, single-cell RNA-seq, and downstream analyses.

Sirius Red/Fast Green staining

Mice were euthanized by CO_2 inhalation. The trachea was surgically exposed, and a cannula or needle connected to an intravenous fluid administration system containing 10% neutral buffered formalin (Fisher Scientific) was gently inserted to preserve lung morphology. The lungs and trachea were carefully dissected from the thoracic cavity to avoid tissue damage and placed in 10% neutral buffered formalin for an additional 48 hours of fixation. Following fixation, tissues were dehydrated in 70% ethanol and embedded in paraffin. Paraffin-embedded lungs were sectioned and stained with hematoxylin and eosin (H&E) at the South Texas Reference Laboratory, UT Health San Antonio. For the assessment of pulmonary fibrosis, Sirius Red/Fast Green staining (MilliporeSigma) was performed on paraffin sections as previously described(2).

Pulmonary Oxygen Saturation and Gas Exchange Testing

Arterial oxygen saturation was measured in conscious mice using the MouseOx Plus Pulse Oximeter (Starr Life Sciences). Whole-body oxygen consumption (VO_2) and carbon dioxide production (VCO_2) were assessed using an open-circuit indirect calorimetry system (Sable Promethion System). Mice were housed individually in metabolic chambers under standard temperature and a controlled light/dark cycle. Measurements were recorded continuously over a 14-day period. Data were analyzed to evaluate oxygen consumption and carbon dioxide excretion.

Single-Cell RNA-seq

Lungs from three mice per experimental condition were harvested and dissociated into single-cell suspensions. Equal amounts of lung tissue from the same lobe of each mouse were pooled and lung single cells were isolated using 10X Genomics GEM-X Flex Sample Preparation kit. Single-

cell libraries were constructed following the 10X Genomics Single Cell Gene Expression Flex protocol and sequenced using an Illumina NovaSeq 6000 platform at the Genome Sequencing Facility at UT Health San Antonio. Raw sequencing data were processed using Cell Ranger (v9.0, 10x Genomics) for alignment to the mouse reference genome (mm10), read counting, and quality control. Putative doublets were identified and removed using DoubletFinder(3). Filtered gene expression matrices were normalized, and highly variable genes were identified using the scran package, then imported into Seurat v5 for log-normalization, scaling, and downstream analyses(4). Batch effects were corrected by applying Harmony to principal component (PCA) embeddings, which were subsequently used for Uniform Manifold Approximation and Projection (UMAP) visualization. A shared nearest-neighbor (SNN) graph was constructed using FindNeighbors, and clusters were identified with FindClusters. Cluster-specific marker genes were determined using FindAllMarkers and used for manual cell-type annotation.

Immunofluorescence staining

Paraffin-embedded tissue sections were deparaffinized, rehydrated, and subjected to heat-induced antigen retrieval in 10 mM sodium citrate buffer containing 0.05% Tween-20 (pH 6.0) at 95°C for 30 min. Sections were then fixed in 10% neutral buffered formalin for 10 min at room temperature and permeabilized with 0.02% Triton X-100 for 5 min. After washing, tissue sections were blocked with 1% normal donkey serum for 30 min and incubated overnight at 4°C with anti-CD68 (clone KP1, Santa Cruz Biotechnology, Cat# sc-20060) or anti-SPP1 (clone E9Z1D, Cell Signaling Technology, Cat# 88742) primary antibodies. After three washes with PBS, sections were incubated with Alexa Fluor 594-conjugated horse anti-mouse IgG (Vector Laboratories, DI-2549) and Alexa Fluor 488-conjugated goat anti-rabbit IgG secondary antibodies (Vector Laboratories,

DI-1488) for 50 min at room temperature. Nuclei were stained with DAPI. Fluorescence images were acquired using Olympus FV-1000 confocal under identical imaging settings for all samples. SPP1-positive macrophages were identified as $CD68^{+}SPP1^{+}$ double-positive cells. For quantitative analysis, the ratio of $CD68^{+}SPP1^{+}$ cells to total $CD68^{+}$ cells were quantified in each field using ImageJ software (NIH). Four randomly selected fields were analyzed per mouse, and the mean value for each mouse was used for statistical analysis.

Sex as a biological variable

Analyzed human dataset includes both male and female subjects. Animal study used male mice. We do not anticipate differences in male and female mice.

Statistics

Data are presented as mean±SD. Replicates are indicated in figure legends. N represents the number of experimental replicates. Statistical comparisons between two groups were performed using unpaired two-tailed Student's *t*-tests. Indirect calorimetry data was analyzed using ANCOVA with body weight as covariate. For single-cell RNA-seq analyses, Wilcoxon rank-sum tests were used for pairwise comparisons. $P < 0.05$ was considered statistically significant.

Study approval: All animal procedures were approved by the Institutional Animal Care and Use Committee (IACUC) of The University of Texas Health Science Center San Antonio.

Data availability: Transcriptomic data are available at GEO database (GSE335923). Values for all data points shown in graphs are provided in the Supporting Data. Other data are available on

reasonable request to the corresponding authors. Analyses were performed using publicly available software and packages as described in Methods, and no custom analytic code was generated for this study.

Author contributions: P.Z. and X.S. conceived the project. X.S., P.Z., J.J. designed the study and performed the experiments. J.J., X.S., W.Y. performed scRNA-seq experiments and bioinformatic analysis. J.J., X.S., and P.Z. wrote the manuscript. The final version was reviewed by all authors.

Funding support: This work was supported by the following:

Cancer Prevention and Research Institute of Texas (CPRIT) RR200089 to PZ

CPRIT RR210005 to XS

Voelcker Young Investigator Award AWD0000144 to PZ.

References

1. Liang J, et al. The ZIP8/SIRT1 axis regulates alveolar progenitor cell renewal in aging and idiopathic pulmonary fibrosis. *J Clin Invest.* 2022;132(11):e157338.
2. Zhao P, et al. An AMPK-caspase-6 axis controls liver damage in nonalcoholic steatohepatitis. *Science.* 2020;367(6478):652-660.
3. McGinnis CS, et al. DoubletFinder: Doublet detection in single-cell RNA sequencing data using artificial nearest neighbors. *Cell Syst.* 2019;8(4):329-337. e324.

4. Hao Y, et al. Dictionary learning for integrative, multimodal and scalable single-cell analysis. *Nat Biotechnol.* 2024;42(2):293-304.
